# Psoriatic arthritis: prospects for the future

**DOI:** 10.1177/1759720X221086710

**Published:** 2022-03-28

**Authors:** Simon Hackett, Alexis Ogdie, Laura C. Coates

**Affiliations:** Nuffield Department of Orthopaedics, Rheumatology and Musculoskeletal Sciences, University of Oxford, Oxford, UK; Division of Rheumatology, Department of Medicine, Perelman School of Medicine, University of Pennsylvania, Philadelphia, PA, USA; Nuffield Department of Orthopaedics, Rheumatology and Musculoskeletal Sciences, University of Oxford, Botnar Research Centre, Windmill Road, Oxford OX3 7LD, UK

**Keywords:** psoriatic arthritis, PsA, future treatment, diagnostic delay

## Abstract

Psoriatic arthritis (PsA) is a form of chronic inflammatory arthritis associated with psoriasis and a multitude of other symptoms, most commonly arthritis, dactylitis, enthesitis and axial involvement. PsA is significantly heterogeneous, with a highly variable clinical course of PsA. Patients may experience significant or mild skin and joint symptoms, with some patients developing rapidly progressing joint destruction and skin symptoms. Despite the range of symptom severity, PsA is frequently associated with significantly impaired quality of life from joint destruction, as well as chronic pain and a range of comorbidities such as depression and cardiovascular disease. Currently, there are no definitive diagnostic tests for PsA, with diagnosis remaining challenging owing to the heterogeneous presentation and course of the disease. Presently, the CASPAR criteria are often used to aid rheumatologists in distinguishing PsA from other inflammatory arthritides. Treatment options for patients have been expanded over the last two decades with the emerging clinical utility of biological therapies. However, early identification and diagnosis of patients and effective disease control remain unmet medical needs within the PsA community. In addition, predicting response to treatment also remains a challenge to rheumatologists. This review highlights the current hurdles faced by healthcare professionals in the diagnosis and management of PsA patients and provides future action points for consideration by the members of the multidisciplinary team who treat PsA patients.

## Introduction

Psoriatic arthritis (PsA) is a heterogeneous condition with musculoskeletal involvement, manifesting as a variety of symptoms including arthritis, dactylitis, enthesitis and axial involvement.^1^ In addition to musculoskeletal symptoms, up to 30% of patients have coexisting psoriasis or nail disease.^2^

PsA was first defined by Moll and Wright in 1970s as ‘an inflammatory arthritis in the presence of psoriasis with a usual absence of rheumatoid factor’.^3^ Despite nearly 50 years passing since the first clinical description, diagnosis remains challenging for clinicians due to lack of validated diagnostic criteria, the inherently heterogeneous nature of the condition and poor identification of the disease, particularly in susceptible patients. Presently, diagnosis relies on identification of clinical signs and symptoms, assessed across multiple domains supported by classification criteria.^4^ Although the developed classification criteria have been well validated in established disease, there is currently an unmet need for early patient identification through the use of diagnostic biomarkers.

Pharmacological treatments for PsA have expanded exponentially over recent years, although long-term therapeutic effects are largely based on clinical experience rather than extensive head-to-head trial-based analysis. Over the last two decades, symptomatic treatments have evolved from traditional disease-modifying antirheumatic agents (DMARDs) to targeted biological therapies.^5^ Although development of biological therapies has revolutionised the treatment of PsA and improved outcomes, predicting and measuring treatment outcomes in patients remains challenging. In addition, there is a growing consensus that identifying and treating patients before the development of clinical features of the disease may be achievable, allowing for the possibility of early intervention, with the goal of disease prevention.

This review will explore the prospects for the future of PsA for both clinicians and patients and provide suggestions around promising efforts to improve diagnosis, disease management and treatment as well as whether the goal of disease prevention is achievable.

## Diagnosis and referral

Globally, the reported prevalence of PsA ranges from 0.3% to 1%^6^ although studies performed worldwide often have significant differences in estimates of prevalence, reflecting a range of methodological differences including variations in classifications used use of incorrect diagnostic coding algorithms and diagnosis using low-sensitivity criteria such as those defined by European Spondyloarthropathy Study Group.^7^ These factors make it challenging to compare meaningful differences in prevalence between studies.

Despite significant advances in the understanding of the pathophysiology of PsA in recent decades, diagnosis remains a challenge. It is estimated that almost 50% of cases in primary and secondary care clinics are unrecognised.^1^ There are no diagnostic criteria available for PsA. In 2006, the ClASsification of Psoriatic ARthritis (CASPAR) classification criteria were developed in 2006 to aid in standardising inclusion of homogeneous population of patients to trials and have been demonstrated extensively to possess both high sensitivity and specificity.^4^ However, classification criteria favour specificity over sensitivity and do not perform as well in diagnosis. Patients often experience a ‘diagnostic odyssey’ with delays in disease identification and prompt referral to secondary care.

Inflammatory markers such as C-reactive protein (CRP) and erythrocyte sedimentation rate (ESR) are normal in approximately 50% of patients.^8^ Presently, no serum biomarkers have been identified with the ability to correlate with diagnosis. A recent study retrospectively analysed serological markers and comorbidities in 629 psoriatic patients, including 102 with PsA.^9^ A range of serological markers were analysed, including anti-extractable nuclear antigens (ENA) autoantibodies, antiphospholipid autoantibodies, and antineutrophil cytoplasmic autoantibodies, as well as haematological and inflammatory parameters. No serological markers were able to distinguish PsA patients, although interestingly, certain comorbidities were more prevalent in the PsA population.

Other studies have examined the potential role of chemokines as diagnostic biomarkers. In 2016, Abji *et al.* reported that levels of CXCL10 are elevated in patients who develop PsA compared with psoriasis patients who do not develop PsA at baseline. In 2020, the same group demonstrated that CXCL10 levels drop following the development of arthritis, with the authors suggesting that their findings warrant further investigation into the predictive value of CXCL10 in PsA diagnosis.^10^

Ultimately, timely diagnosis and early intervention are critical in PsA, with studies showing that aggressive targeted approach to treating psoriatic arthritis greatly improves disease-activity outcomes for patients, reducing long-term disability and long-term join damage.^11^

### Early identification of patients

The majority of PsA patients present with a heterogeneous picture of the disease which may include skin and nail involvement, dactylitis, enthesitis, spondylitis and arthritis.^12^ As outlined earlier, identification and subsequent diagnosis of PsA is based on clinical findings rather than strict biochemical or radiological findings, often making identification challenging.

The first step on the PsA patient journey is often self-identification of symptoms. The majority of patients with PsA also have pre-existing psoriasis; however, studies have shown that there are many cases of established PsA which remain unidentified for some time, despite an established diagnosis of psoriasis.^1^ It has been suggested that the lack of an established diagnosis may result from poor understanding of the link between the skin and arthritis, lack of rheumatological education among people with psoriasis, primary care physicians (PCPs) and treating dermatologists.^1^ To help drive early diagnosis in this ‘at-risk’ patient group, recent guidance by National Institute for Health and Care Excellence (NICE) for management of psoriasis recommends annual screening for PsA among patients with psoriasis both in primary and secondary care settings.^13^ Alongside guidance for healthcare professionals (HCPs), attempts to improve screening, such as the distribution of educational material to psoriasis patients, may help improve screening attendance.^1^ Targeting the time point within their psoriasis journey at which patients should be screened is also of importance. A recent study identified that physician associates and nurse practitioners in dermatology clinics and primary care practices are often first to see patients with psoriasis and are therefore ideally positioned to screen them for PsA and refer them to a rheumatologist as needed.^14^ Harnessing the ability for HCPs to screen for PsA in susceptible patients is an important approach to take to ensure timely referral and effective early disease treatment.

Although screening is a potentially useful tool for the identification of PsA patients, it is likely to be only restricted to patients with psoriasis. The complex symptomatology of PsA means that identification of PsA by PCPs is often low, with a recent Multinational Assessment of Psoriasis and Psoriatic Arthritis survey of 391 dermatologists and 390 rheumatologists based in North America and Europe, > 75% stating that PsA is probably underdiagnosed due to a failure to connect skin and joint symptoms.^15^ To address the potential educational deficits among PCPs and other HCPs, it has been suggested that health authorities and academic societies should create educational awareness campaigns aimed at PCPs and dermatologists about the symptoms of PsA to improve understanding of the disease.^16^

### Improving referral and diagnostic pathways for PsA

As outlined earlier, early diagnosis is key to improving outcomes in PsA patients as it allows prompt, aggressive, targeted treatment with anti-inflammatory disease-modifying drugs such as methotrexate or biologics results in reduced progression of joint damage. Indeed, the 2018 American College of Rheumatology/National Psoriasis Foundation Guideline for the Treatment of PsA states that early commencement of therapy is critical for improving long-term outcomes in patients, suggesting a key window of opportunity exists for diagnosis and intervention in PsA patients.^17^

However, despite the overwhelming evidence for the importance of early referral, delayed referral and subsequent diagnostic delay is common among inflammatory arthritides, including PsA. A recent study examined the diagnostic delay in PsA patients using data from the National Clinical Audit for Rheumatoid and Early Inflammatory Arthritis, undertaken by the British Society for Rheumatology. Analysis demonstrated that patients with PsA had a significantly longer delay to presentation and diagnosis than those with rheumatoid arthritis (RA), with a mean time to referral of 5.4 weeks following consultation with their general practitioner (GP), compared with 4.0 weeks for RA patients.^18^

In order to help reinforce the importance of early referral, several measures have been proposed to help reduce diagnostic delay. Guidelines, such as European League Against Rheumatism (EULAR) standard of care, have been developed to help encourage patients to be referred early to a rheumatologist by their PCP if PsA is suspected.^19^ In addition to standard of care guidance, multidisciplinary care is important for prompt referral of patients. A recent 12-point recommendation framework suggested that improved collaboration between dermatologists, PCPs and rheumatologists may hold the key to reducing time to PsA diagnosis. The authors suggested that this may take the form of standard referral pathways, multidisciplinary team meetings, or ‘one stop’, rapid-access combined clinics in which the patient is seen by multiple specialists at the same time.^20^ To help facilitate these referral pathways, a variety of screening tests such as Psoriatic arthritis UnclutteRed screening Evaluation (PURE-4),^21^ Psoriatic Arthritis Screening and Evaluation (PASE)^22^ and the Psoriasis Epidemiology Screening Tool (PEST)^23^ have been validated, which may aid clinicians in expediting referrals for at-risk patients.

Although diagnostic delay remains an issue for PsA patients, a study published in 2015 demonstrated that between 2000 and 2011, a significant reduction in diagnostic delay was observed in inflammatory arthritis patients, including PsA in Denmark through analysis of the DANBIO registry.^24^ Although this suggests that there may be an emerging stronger awareness of the importance of early diagnosis in PsA, the findings were within one country, and in healthcare systems which have lower levels of integration across specialities, these observations may not translate to other populations. It is therefore of importance that combined clinics between dermatologists and rheumatologists focus on screening psoriasis patients and this may in turn drive earlier diagnosis.

**Action points:**

1. Encourage regular screening and education of at-risk patients such as those with psoriasis
2. Ensure educational opportunities are tailored towards localised referral and diagnostic pathways
3. Promote disease awareness and collaboration among HCPs including PCPs, dermatologists and other allied HCPs.

## Treatment and management of PsA

Over the last 20 years, therapeutic options for rheumatological conditions such as PsA have evolved at a considerable pace. Over the last decade, treatment has shifted away from traditional DMARD drugs such as methotrexate towards the development of biological therapies such as tumour necrosis factor (TNF) inhibitors, interleukin (IL)-12 and IL-23 inhibitors and IL-17 inhibitors, which have proved highly efficacious in a range of clinical trials.^25–27^ Choice of treatment varies according to guidance: EULAR recommends the use of TNF inhibitors, ustekinumab and IL-17 inhibitors for peripheral arthritis which is unresponsive to DMARDs.^28^ The treatment recommendations by EULAR help support treatment decision making and address the spectrum of disease phenotypes seen in PsA patients. As the authors note, however, the guidelines will need to be updated regularly, in light of emerging data following treatment of patients.

In contrast to EULAR, the ACR guidelines recommend first-line treatment with a TNF inhibitor over oral small molecule DMARDs in order to ‘treat to target’.^17^ The guidelines by ACR suggest this approach as early treatment with TNF inhibitors could delay or avoid the irreversible joint damage seen in PsA patients, supporting an overall improvement in quality of life (QoL).

In addition to the EULAR and ACR guidelines for treatment, the Group for the Research and Assessment of Psoriasis and Psoriatic Arthritis (GRAPPA) developed treatment recommendations in 2015. These were updated in 2021, following emergence of new treatment data and therapeutics.^29^ The authors suggested considering which domains are involved, as well as patient preference and any previous or concomitant therapies. Furthermore, the choice of therapy should address treatment across as many domains as possible (peripheral arthritis, axial disease, enthesitis, dactylitis, skin and nails). Alongside these factors, comorbidities and any other associated conditions should be considered which may impact the choice of therapy. Patients should be periodically re-evaluated following commencement of treatment and therapy modified as required.

However, despite a range of treatment options and durable efficacy among therapies, along with carefully considered guidelines, predicting response to treatment remains an issue yet to be addressed. Furthermore, it remains unclear why certain treatments fail to adequately control disease in certain patients. Alongside predicting treatment responses, ascertaining and implementing nonpharmacological management of PsA patients remains a priority for the future.

## Predicting treatment response: a role for precision medicine?

Precision medicine is defined as ‘an emerging approach for disease treatment and prevention that considers individual variability in genes, environment, and lifestyle for each person’.^30^ Although precision medicine has been applied to other areas of disease, for example, determining Her2 status in breast cancer patients, the use of precision medicine in rheumatology remains very much in its infancy.

PsA presents a unique opportunity for driving a rationalised target-driven therapeutic approach through the application of personalised medicine. A plethora of studies have attempted to elucidate the immunological components underpinning PsA, which has helped drive the development of such therapies as ustekinumab.^31^ Although patients demonstrate common immunological dysregulation, such as overt Th17 activation, the individual immunophenotype of each patient is unique and driven by a range of genetic, environmental and tissue-specific differences.^32^ It is likely, therefore, that the individual immunophenotype influences response to treatments. This influence has been demonstrated in a study examining the use of immunophenotyping in guiding choice of biological therapy in PsA patients.^33^ In the study of 64 PsA patients, half of the patients received lymphocyte phenotyping which guided treatment towards ustekinumab for patients with activated Th1-dominance status, secukinumab for patients with activated Th17-dominance status and adalimumab (ADA) or infliximab (IFX) for patients with activated Th1/Th17-high status. The other half of the patients were managed according to physician or patient preference of biologic. Disease activity was assessed in all patients using simplified disease activity index (SDAI) as well as psoriasis area and severity index. After 6 months, the rate of low disease activity achievement according to SDAI at 6 months was significantly higher in the strategic biological treatment group compared with that of the physician/patient preference biological treatment group. These optimistic findings suggest that further elucidation of the pathways involved in immune dysregulation in PsA patients may allow for the use of immunophenotyping in helping guide appropriate treatment, through the sampling of peripheral blood. Although this offers a potentially attractive approach to personalised treatment in PsA patients, whether immunophenotyping can reflect disease severity accurately in a range of tissues remains to be ascertained. In order to further characterise any potential correlations, other disease areas such as RA have explored the use of collaborative approaches such as the formation of Maximizing Therapeutic Utility in RA (MATURA) consortium.^34^ A similar approach in PsA may help determine the real-world analysis of peripheral blood collected from large cohorts of patients, who are then followed to assess response in order to identify immunophenotypes predictive of response, alongside other factors that correlate with treatment outcomes.

Alongside predictive measures for forecasting treatment response, a range of novel therapies are currently under development

### Holistic PsA patient management: beyond pharmacology

Aside from pharmacological treatments, there is an increasing wealth of evidence to suggest that holistic disease management in rheumatology patients is important, particularly with respect to managing QoL and psychosocial burden associated with PsA. The role of other HCPs in managing PsA patients spans a variety of domains including multidisciplinary care, psychological management and pain management.

Current guidelines issued by EULAR approach the management of PsA primarily from the perspective of a rheumatologist.^19^ However, it is well recognised that other HCPs including primary care physicians and dermatologists play an important role in the treatment and management of PsA patients. Models for integrating multidisciplinary management have been described elsewhere, including dual rheumatologist–dermatologist assessment, as well as parallel and circuit models of patient review.^35^ Alongside management of joint complaints and skin symptoms, it has been shown that the comorbidity burden among PsA patients is significantly higher than the general population, with an increased prevalence of hyperlipidaemia, hypertension and inflammatory bowel disease.^36^ Compared with the general population, patients with PsA have a 55% increased risk of developing a cardiovascular event, with significantly higher prevalence of myocardial infarctions, cerebrovascular diseases, and heart failure in patients.^37^ Furthermore, patients with PsA appear to have a higher cardiovascular risk compared with patients with psoriasis alone. It has been hypothesised that the chronic inflammatory state characteristic of PsA contributes to the increased comorbidity burden observed in patients.^37^

The multisystemic nature of PsA therefore requires patients are managed and cared for by multiple specialities. The evolving model of multidisciplinary care is likely to incorporate a wider integration of HCPs than previously constructed guidelines. It should be remembered, however, approaches will likely be dictated by local healthcare systems and resource allocations.^35^

PsA is associated with a considerable psychosocial burden. Indeed, previous studies have shown that not only do PsA patients have considerably poorer QoL compared with the general population, but QoL and functional status is also considerably worse compared with patients with psoriasis or RA.^38^ A recent study suggested that treatment of PsA and associated pain cannot be effectively achieved without addressing all psychosocial factors, including simultaneous management of physical and psychological concerns.^39^ Clearly the former may be addressed by the treating dermatologist and rheumatologist, with the latter evaluated by a psychologist. Furthermore, a cross-sectional study carried out across 131 PsA outpatient clinics showed fatigue, sleep disturbances, anxiety/depression, impaired physical function, unemployment and presence of comorbidities were independently associated with impaired health-related QoL (HRQoL) in patients.^40^

Alongside acknowledging this burden faced by patients, an important question that rheumatologists should be aware of is what role, if any, does the proinflammatory environment contribute to the greater psychosocial burden seen in PsA patients? Studies have demonstrated that a range of inflammatory cytokines, including IL-6 and IL-12, play a role in the development of depression.^41^ Early aggressive treatment in patients with PsA patients targeting key cytokines involved in the neuroinflammatory components of depression may prove a potential course of action in managing mood changes. Of note, in other inflammatory arthritides, such as RA, longer standing depressive symptoms correlate with reduced treatment responses with respect to achieving disease control.^42^ These findings have also been noted in a prospective multicentre study in PsA patients in Norway, with depression and anxiety reducing the likelihood of joint remission following treatment.^43^

Extrapolating this study to PsA patients further lends weight to the argument that addressing any mood changes early on following diagnosis should be a priority in providing holistic care to patients. Ultimately, these findings highlight the importance of psychosocial management of patients and, importantly, given the chronic nature of PsA, suggest patients should be regularly evaluated, in particular, following any increase in disease activity.

Despite the prevalence of pain in PsA patients, historically, therapeutic trials did not routinely report pain specifically as an outcome, although more recent trials, such as the FUTURE 2 study, have demonstrated that secukinumab treatment offers a significant and sustained reduction in pain over a 2-year period.^44^ Even with the development and use of both DMARDs and biological therapies, persistent pain is often a significant issue for PsA patients. In a study by EULAR, a questionnaire, Psoriatic Arthritis Impact of Disease (PsAID), identified pain as the most important health domain affecting HRQoL.^28^ Around a third of PsA patients receiving biological therapies report no or mild pain, with a third reporting moderate pain and a further third describing severe pain.^45^ Pain is clearly common among PsA patients receiving treatment and the more severe the pain, the greater impact on physical functioning, work productivity and engagement in activities.^45^

Even in light of improved pain symptoms in treated PsA patients, speedy, effective and long-term management is required to improve QoL for patients. Prompt referral to pain specialists for the treatment of refractory or difficult to manage pain should be considered under the umbrella of multidisciplinary management of PsA patients. In order to help rationalise pain management approaches in patients, further studies are required to draft consensus guidelines on optimum pain management strategies in PsA patients.

**Action points:**

1. Implementation of multidisciplinary management of PsA patients should remain a priority in all rheumatology departments
2. Consider regularly incorporating psychological outcomes and pain management into clinical trial designs
3. Consider prompt referral to pain specialists if pain remains uncontrolled
4. Guidelines on optimum pain management in PsA patients should be developed using emerging evidence from recent trials

## Assessing disease activity in PsA

The assessment of disease activity is pivotal in guiding treatment. Over recent years, a range of composite scoring systems have been developed in order to accurately and reliably assess disease. However, despite the creation of assessment tools, such as Minimal Disease Activity (MDA), Psoriatic Arthritis Disease Activity Score (PASDAS) and Disease Activity Index for Psoriatic Arthritis (DAPSA), the inherent heterogeneity of PsA makes translating such symptoms into a validated relevant for all measure challenging.^46,47^ Given this, developing novel tools to assess disease activity through biomarkers and technological based approaches is a subject of keen interest within the field. These assessments can then also help in directing care and act as a triage tool; for example, whether the patient needs to be seen promptly or whether they can be seen at a later timepoint.^48^

### Disease biomarkers

Given the clinical heterogeneity of PsA, potential biomarkers have long been sought by rheumatologists that are reflective of treatment response.

With respect to treatment response, a range of potential biomarkers have been suggested including synovial CD3^+^ cell number,^49^ C-reactive protein^50^ and matrix metalloprotease-3^51^ to name only a few. A recent systematic review of treatment response biomarkers suggested that CRP and subsequent response to biological therapy is potentially of significant clinical utility, although the studies examined only patients treated with anti-TNF therapy.^52^

The ability for rheumatologist to accurately diagnose and predict treatment responses in PsA patients remains an unmet medical need which warrants careful consideration in future clinical trials.

### Technological-based methods

As rheumatology clinics become increasingly ‘digitally mature’, the development and integration of technology to support self-monitoring and self-management has expanded dramatically.^53^ Such technologies provide a unique opportunity to not only help monitor and guide treatment in patients, but also help collect real-world evidence (RWE) of long-term outcomes in treated patients.

The use of digital technology has already been examined in patients with RA in a range of applications including reporting symptoms prior to clinic attendance, remote monitoring, symptom tracking and racking symptoms through increased insight into changes in their disease through time.^53^

**Figure 1.**
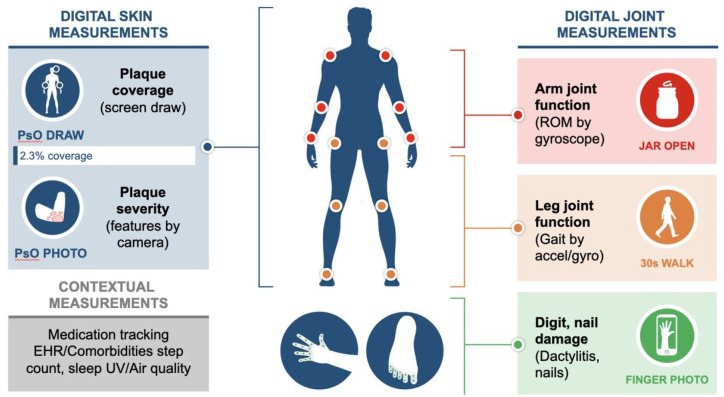
Psorcast is a PPACMAN–Sage collaborative venture with the aim of creating tailored forecast of disease, along with appropriate management by capturing digital skin and joint measurements through smartphone technology.

The COVID-19 pandemic has presented significant challenges to both rheumatologists and patients alike. Along with the direct consequences of the pandemic, the management and follow-up of patients has been severely impacted due to a range of factors including social restrictions, travel restrictions and re-deployment of HCPs. Considering the challenges faced by clinicians following up PsA patients, the use of smartphone sensor technology has been explored as a tool for quantitatively measuring disease symptoms in patients. Recently, three novel smartphone sensor-based measurement tools were developed as part of *Psorcast* to assess PsA symptoms affecting joints domains as shown in Figure 1. The *Digital Jar Open* tool uses the gyroscope to measure inward and outward rotation of each arm to generate an inward symmetry score and outward symmetry score that are normalised within each participant. The *30-Second Walk* tool measures gait with the smartphone in a pocket during a walk to generate a symmetry score using PDKit. The *Finger/Toe Photo* captures finger and toe images, normalising them with the contralateral nail bed width to measure relative digit widths. Assessment of this novel tool in the patients recruited so far has demonstrated that the three sensor-based measurements can distinguish some clinical features of PsA. Although further validation is required, these and other *Psorcast* tools may provide for remote self-assessment when clinical visits cannot be performed. Importantly, longitudinal and frequent symptom measurement could be of high value to study disease progression and assessing treatment response.^54^

In addition to tools such as Psorcast, the use of artificial intelligence (AI) has also been proposed as a tool to help predict disease progression, flares and ‘at risk’ patients who have a higher propensity to develop PsA on a background of psoriasis.^55^ Indeed, in 2019, EULAR published a range of points for developers and HCPs to consider when evaluating the implementation of mobile health applications in rheumatological patients.^56^

Alongside AI, the use of mHealth, as defined by the World Health Organization (WHO) as ‘the use of mobile and wireless technologies to support the achievement of health objectives’ may also prove useful in encouraging self-management of disease in patients with PsA. The potential of mHealth in managing patients has been reviewed by Fagni *et al.*,^57^ and although the authors are optimistic about the potential of mHealth uptake in PsA patients, several barriers to successful implementation remain, including poor levels of technological literacy among older patients, a lack of high-quality apps in terms of scientific accuracy and compliance to evidence-based guidelines.

**Action points:**

1. The clinical relevance of biomarkers should be further established in larger, more well-defined cohorts
2. Further validation of novel technological tools for determining disease activity and patient outcomes should continue
3. Consider the implementation of artificial intelligence for prediction and forecast of disease progression and symptoms

## Moving towards disease prevention

Although a significant amount of effort has been placed on the management and treatment of PsA, there is growing evidence to suggest that targeting patients who are at an increased risk of developing PsA may be amenable to interventions that slow disease onset or even prevent disease. The questions are therefore clear: which patients are at risk of developing PsA and how may disease progression be prevented?

Over recent years, the theory that psoriasis and PsA are in fact overlapping conditions, both of which are underpinned by a proinflammatory environment, has gained considerably traction. As discussed earlier, there has been a long and well-demonstrated link that psoriasis is a strong risk factor for PsA, with up to 30% of patients with psoriasis developing inflammatory synovio-entheseal manifestations.^58^ Furthermore, psoriasis often precedes inflammatory joint involvement by an average of 7 years, suggesting there is ample time for intervention.^58^ Within this patient population, a range of risk factors have been shown to suggest an increased risk of the development of PsA including a range of major histocompatibility complex (MHC) mutations, such as HLA-Cw*0602, HLA-B27, HLA-B38, HLA-B39, as well as non-MHC mutations, increased body mass index (BMI) and bodily distribution of psoriasis and severity.^59^ Current analysis of available data has, however, failed to find a single variable which adequately predicts transition to synovio-entheseal disease.

Reflecting on disease stages apparent in PsA may also offer insight for how to target patients at risk of developing PsA. A recent Delphi consensus study has aimed to help define specific subgroups of individuals during early preclinical and clinical phases of PsA for use in research studies.^60^ Following a three-round Delphi process, consensus was reached for three terms and definitions: ‘increased risk for PsA’, ‘psoriasis with asymptomatic synovio-entheseal imaging abnormalities’ and ‘psoriasis with musculoskeletal symptoms not explained by other diagnosis’. It is hoped that identification of these terms will allow for more well-defined populations in studying patients who may have an increased risk of developing PsA.

A recent systematic literature review and meta-analysis examined a range of predictors of PsA development in psoriasis patients.^61^ The authors identified 26 articles that were deemed suitable for inclusion and analysis. Psoriasis patients with arthralgia and imaging-musculoskeletal inflammation were at high risk of developing PsA, along with increased body mass index (BMI) and a family history of PsA. These findings may prove useful for helping to identify PsA in its preclinical phase and will potentially allow for the design of trials aiming to prevent development of PsA.

The role of treatment and development of PsA has also been studied in psoriasis patients. A retrospective nonrandomised study in patients with moderate-to-severe plaque psoriasis who were prescribed  > 5 years of biologic DMARD therapy were assessed for development of PsA and the annual and cumulative incidence rates analysed.^62^ The authors demonstrated that biologic DMARDs may delay or reduce the risk of incident PsA in moderate-to-severe plaque psoriasis patients, suggesting that treatment modality may play an important role in long-term risk. Further to this study, a retrospective cohort study examined 193,709 patients with psoriasis but without PsA. The authors demonstrated that biologic use was associated with the development of PsA among patients with psoriasis although acknowledged this may have been related to confounding by indication and protopathic bias.^63^ It is therefore clear that further studies, in particular those that are prospective in nature, are required to further elucidate the relationship between risk and PsA development.

Along with underlying risk factors, the transition from psoriasis to PsA is likely an interplay between genes, immunity and the environment and has been proposed to evolve through stages.^58,64^ The proposed transition includes establishment of a proinflammatory environment alongside psoriasis through the interaction of genetic and environmental factors. The preclinical phase includes activation of the IL-23-IL-17 axis alongside TNF-α production. Following this, a subclinical phase is apparent with appearance of soluble biomarkers and emergence of to synovio-enthesitis, shortly followed by a prodromal phase of arthralgia. The final phase results in clinically evident PsA with classical symptoms such as synovitis, enthesitis, dactylitis and asymmetric axial disease.^65^ It is clear, therefore, that early identification of patients before they progress past the preclinical phase of psoriasis–PsA disease evolution. Early identification of these patients remains challenging, although the expanding role of identifiable biomarkers, stratified to risk of disease progression, remains an active area of interest in PsA patients. Although there are currently no validated biomarkers, high baseline serum concentrations of CXC-chemokine ligand 10 (CXCL10) in patients with psoriasis correlate with risk of developing PsA.^10^ Furthermore, a number of other potentially clinically relevant biomarkers including M2BP and ITGB5 show potential promise in aiding clinicians identify patients at risk of disease progression.^66^ In addition to biomarker identification, use of imaging modalities such as ultrasound and magnetic resonance imaging may help detect patients with silent joint disease, although the predictive ability of detection of such changes remains unknown. Although identification and screening for PsA in psoriasis patients should remain an important focus, this approach relies on patients presenting with skin disease prior to join symptoms, which is not the case for all patients. The subset of patients without skin disease may require alternative approaches to ensure early diagnosis. In addition, developing a predictive tool that utilises data from psoriasis patients who may be at risk of developing PsA may aid in the design of preventive trials.^58^

**Action points:**

1. Elucidation of biomarkers to predict patients at risk of disease progression should be prioritised
2. Establishment of dialogue between patients and HCPs to ascertain the level of disease severity before treatment should be commenced to ensure a balance of treatment benefit and any potential risks
3. Continuing to further define specific subgroups of individuals during early preclinical and clinical phases of PsA for analysis in preventive research studies
4. Harness a range of methods to communicate disease education to at-risk patient populations
5. Develop designs for interventional studies to prevent or delay PsA development

## Conclusion and future directions

It is clear that the understanding and treatment of PsA has evolved rapidly over recent years. Despite the rapid advancements outlined in this review, there are still a range of clear unmet medical needs within the PsA community. In particular, the ability to identify disease early and facilitate rapid access to treatment is a key priority for both clinicians and patients.

Although there have been a variety of efforts over recent years to address these challenges, progress and implementation has been slow, often accompanied by unrealistic expectations. We believe that the PsA community is now at a precipice: Now is a time to pause, reflect and consolidate ideas and pursue the most appropriate avenues to explore in order to achieve optimum patient care and outcomes.

It is likely that over the next decade, our knowledge of disease assessment and prediction of disease in patients will shift rapidly, in conjunction with the application of personalised medicine and biomarkers. Although these tests will undoubtedly help to guide diagnosis and treatment, they still remain a significant challenge to develop and validate from both clinical and financial perspectives.

Ultimately, even in the absence of no significant changes in treatment efficacy, it is hoped that we should be at a point where patient outcome and QoL is better simply because the rheumatology community has learnt how to manage and treat patients from a more holistic and integrated approach.

## References

[1] Coates LC Helliwell PS . Psoriatic arthritis: state of the art review. Clin Med (Lond) 2017; 17: 65–70. 28148584 10.7861/clinmedicine.17-1-65 PMC6297592

[2] FitzGerald O Ogdie A Chandran V , et al. Psoriatic arthritis. Nat Rev Dis Primers 2021; 127: 59. 10.1038/s41572-021-00293-y 34385474

[3] Moll JM Wright V . Psoriatic arthritis. Semin Arthritis Rheum 1973; 3: 55–78. 4581554 10.1016/0049-0172(73)90035-8

[4] Taylor W Gladman D Helliwell P , et al. Classification criteria for psoriatic arthritis: development of new criteria from a large international study. Arthritis Rheum 2006; 54: 2665–2673. 16871531 10.1002/art.21972

[5] D’Angelo S Tramontano G Gilio M , et al. Review of the treatment of psoriatic arthritis with biological agents: choice of drug for initial therapy and switch therapy for non-responders. Open Access Rheumatol 2017; 9: 21–28. 28280401 10.2147/OARRR.S56073 PMC5338946

[6] Gladman DD Antoni C Mease P , et al. Psoriatic arthritis: epidemiology, clinical features, course, and outcome. Ann Rheum Dis 2005; 64: ii14–ii17. 10.1136/ard.2004.032482 PMC1766874 15708927

[7] Taylor WJ Marchesoni A Arreghini M , et al. A comparison of the performance characteristics of classification criteria for the diagnosis of psoriatic arthritis. Semin Arthritis Rheum 2004; 34: 575–584. 15609261 10.1016/j.semarthrit.2004.05.001

[8] Punzi L Podswiadek M Oliviero F , et al. Laboratory findings in psoriatic arthritis. Reumatismo 2007; 59: 52–55. 17828345 10.4081/reumatismo.2007.1s.52

[9] Su YJ . Early diagnosis of psoriatic arthritis among psoriasis patients: clinical experience sharing. Clin Rheumatol 2020; 39: 3677–3684. 32468320 10.1007/s10067-020-05132-1 PMC7648743

[10] Abji F Pollock RA Liang K , et al. Brief report: CXCL10 is a possible biomarker for the development of psoriatic arthritis among patients with psoriasis. Arthritis Rheumatol 2016; 68: 2911–2916. 27389865 10.1002/art.39800

[11] Haroon M Gallagher P FitzGerald O . Diagnostic delay of more than 6 months contributes to poor radiographic and functional outcome in psoriatic arthritis. Ann Rheum Dis 2015; 74: 1045–1050. 24525911 10.1136/annrheumdis-2013-204858

[12] Coates LC Strand V Wilson H , et al. Measurement properties of the minimal disease activity criteria for psoriatic arthritis. RMD Open 2019; 5: e001002. 10.1136/rmdopen-2019-001002 PMC6744081 31565243

[13] NICE Guidance. Psoriasis. QS40, https://www.nice.org.uk/guidance/qs40

[14] Mease PJ Gladman DD Helliwell P , et al. Comparative performance of psoriatic arthritis screening tools in patients with psoriasis in European/North American dermatology clinics. J Am Acad Dermatol 2014; 71: 649–655. 24974240 10.1016/j.jaad.2014.05.010

[15] van de Kerkhof PC Reich K Kavanaugh A , et al. Physician perspectives in the management of psoriasis and psoriatic arthritis: results from the population-based Multinational Assessment of Psoriasis and Psoriatic Arthritis survey. J Eur Acad Dermatol Venereol 2015; 29: 2002–2010. 25885420 10.1111/jdv.13150 PMC5029592

[16] Betteridge N Boehncke WH Bundy C , et al. Promoting patient-centred care in psoriatic arthritis: a multidisciplinary European perspective on improving the patient experience. J Eur Acad Dermatol Venereol 2016; 30: 576–585. 26377041 10.1111/jdv.13306 PMC5049610

[17] Singh JA Guyatt G Ogdie A , et al. Special article: 2018rican College of Rheumatology/National Psoriasis Foundation Guideline for the Treatment of Psoriatic Arthritis. Arthritis Rheumatol 2019; 71: 5–32. 30499246 10.1002/art.40726 PMC8218333

[18] Holland R . Psoriatic arthritis is associated with diagnostic delay and worse outcome at three months when compared to rheumatoid arthritis. Ann Rheum Dis 2017; 76: 685.

[19] Gossec L Baraliakos X Kerschbaumer A , et al. EULAR recommendations for the management of psoriatic arthritis with pharmacological therapies: 2019ate. Ann Rheum Dis 2020; 79: 700–712. 32434812 10.1136/annrheumdis-2020-217159 PMC7286048

[20] GratacÃ³s J Behrens F Coates LC , et al. A 12-point recommendation framework to support advancement of the multidisciplinary care of psoriatic arthritis: a call to action. Joint Bone Spine 2021; 88: 105175. 33771760 10.1016/j.jbspin.2021.105175

[21] Audureau E Roux F Lons Danic D , et al. Psoriatic arthritis screening by the dermatologist: development and first validation of the ‘PURE-4 scale’. J Eur Acad Dermatol Venereol 2018; 32: 1950–1953. 29430720 10.1111/jdv.14861

[22] Torrente-Segarra V Reina D Roig D , et al. AB0830cordance of the Pase Questionnaire (Psoriatic Arthritis Screening Evaluation) for the screening and assessment of clinical practice in psoriatic arthritis. Ann Rheum Dis 2015; 74: 1178.

[23] Helliwell PS . Psoriasis epidemiology screening tool (PEST): a report from the GRAPPA 2009ual meeting. J. Rheumatol 2011; 38: 551–552. 21362785 10.3899/jrheum.101119

[24] Sørensen J Hetland ML ; and all departments of rheumatology in Denmark. Diagnostic delay in patients with rheumatoid arthritis, psoriatic arthritis and ankylosing spondylitis: results from the Danish nationwide DANBIO registry. Ann Rheum Dis 2015; 74: e12. 10.1136/annrheumdis-2013-204867 PMC4345887 24534758

[25] Mease PJ Goffe BS Metz J , et al. Etanercept in the treatment of psoriatic arthritis and psoriasis: a randomised trial. Lancet 2000; 356: 385–390. 10972371 10.1016/S0140-6736(00)02530-7

[26] McInnes IB Kavanaugh A Gottlieb AB , et al. Efficacy and safety of ustekinumab in patients with active psoriatic arthritis: 1 year results of the phase 3, multicentre, double-blind, placebo-controlled PSUMMIT 1 trial. Lancet 2013; 382: 780–789. 23769296 10.1016/S0140-6736(13)60594-2

[27] McInnes IB Sieper J Braun J , et al. Efficacy and safety of secukinumab, a fully human anti-interleukin-17A monoclonal antibody, in patients with moderate-to-severe psoriatic arthritis: a 24-week, randomised, double-blind, placebo-controlled, phase II proof-of-concept trial. Ann Rheum Dis 2014; 73: 349–356. 23361084 10.1136/annrheumdis-2012-202646

[28] Gossec L de Wit M Kiltz U , et al. A patient-derived and patient-reported outcome measure for assessing psoriatic arthritis: elaboration and preliminary validation of the Psoriatic Arthritis Impact of Disease (PsAID) questionnaire, a 13-country EULAR initiative. Ann Rheum Dis 2014; 73: 1012–1019. 24790067 10.1136/annrheumdis-2014-205207

[29] Coates LC Soriano E Corp N , et al. OP0229 Group For Research And Assessment Of Psoriasis And Psoriatic Arthritis (Grappa) Treatment Recommendations 202. Ann Rheum Dis 2021; 80: 139–140.

[30] Raja K Dasot N Goyal P , et al. Towards evidence-based precision medicine: extracting population information from biomedical text using binary classifiers and syntactic patterns. AMIA Jt Summits Transl Sci Proc 2016; 2016: 203–212. 27570671 PMC5001749

[31] Dobbin-Sears I Roberts J O’Rielly DD , et al. Ustekinumab in psoriatic arthritis and related phenotypes. Ther Adv Chronic Dis 2018; 9: 191–198. 30263103 10.1177/2040622318781760 PMC6151900

[32] Naik GS Ming WK Magodoro IM , et al. Th17 inhibitors in active psoriatic arthritis: a systematic review and meta-analysis of randomized controlled clinical trials. Dermatology 2017; 233: 366–377. 29258093 10.1159/000484520

[33] Miyagawa I Nakayamada S Nakano K , et al. Precision medicine using different biological DMARDs based on characteristic phenotypes of peripheral T helper cells in psoriatic arthritis. Rheumatology (Oxford) 2019; 158: 336–344. 10.1093/rheumatology/key069 29618121

[34] Barton A Pitzalis C . Stratified medicine in rheumatoid arthritis-the MATURA programme. Rheumatology (Oxford) 2017; 56: 1247–1250. 28165532 10.1093/rheumatology/kew369 PMC5850849

[35] Visalli E Crispino N Foti R . Multidisciplinary management of psoriatic arthritis: the benefits of a comprehensive approach. Adv Ther 2019; 36: 806–816. 30805821 10.1007/s12325-019-00901-0

[36] Haddad A Zisman D . Comorbidities in patients with psoriatic arthritis. Rambam Maimonides Med J 2017; 8: e0004. 10.5041/RMMJ.10279 PMC5298365 28178440

[37] Perez-Chada LM Merola JF . Comorbidities associated with psoriatic arthritis: review and update. Clin Immunol 2020; 214: 108397. 32229290 10.1016/j.clim.2020.108397

[38] Husted JA Gladman DD Farewell VT , et al. Health-related quality of life of patients with psoriatic arthritis: a comparison with patients with rheumatoid arthritis. Arthritis Rheum 2001; 45: 151–158. 11324779 10.1002/1529-0131(200104)45:2<151::AID-ANR168>3.0.CO;2-T

[39] Husni ME Merola JF Davin S . The psychosocial burden of psoriatic arthritis. Semin Arthritis Rheum 2017; 47: 351–360. 28802776 10.1016/j.semarthrit.2017.05.010

[40] Haugeberg G Michelsen B Kavanaugh A . Impact of skin, musculoskeletal and psychosocial aspects on quality of life in psoriatic arthritis patients: a cross-sectional study of outpatient clinic patients in the biologic treatment era. RMD Open 2020; 6: e001223. 10.1136/rmdopen-2020-001223 PMC7299507 32409518

[41] Himmerich H Patsalos O Lichtblau N , et al. Cytokine research in depression: principles, challenges, and open questions. Front Psychiatry 2019; 10: 30. 30792669 10.3389/fpsyt.2019.00030 PMC6374304

[42] Matcham F Davies R Hotopf M , et al. The relationship between depression and biologic treatment response in rheumatoid arthritis: an analysis of the British Society for Rheumatology Biologics Register. Rheumatology (Oxford) 2018; 157: 835–843. 10.1093/rheumatology/kex528 29447376

[43] Michelsen B Kristianslund EK Sexton J , et al. Do depression and anxiety reduce the likelihood of remission in rheumatoid arthritis and psoriatic arthritis? Data from the prospective multicentre NOR-DMARD study. Ann Rheum Dis 2017; 76: 1906–1910. 28733473 10.1136/annrheumdis-2017-211284

[44] McInnes IB Mease PJ Schett G , et al. Secukinumab provides rapid and sustained pain relief in psoriatic arthritis over 2 years: results from the FUTURE 2 study. Arthritis Res Ther 2018; 720: 113. 10.1186/s13075-018-1610-3 PMC5992664 29880010

[45] Conaghan V Strand R Alten R , et al. Pain still remains a high unmet need among psoriatic arthritis patients receiving existing biologic treatment: results from a multi national real-world survey, EULAR 2017. Abstract: OP0107. Ann Rheum Dis 2017; 76: 96–97.27165179

[46] Hackett S Coates L . Psoriatic arthritis: an up to date overview. Ind J Rheum 2020; 15: 45–51.

[47] Schoels MM Aletaha D Alasti F , et al. Disease activity in psoriatic arthritis (PsA): defining remission and treatment success using the DAPSA score. Ann Rheum Dis 2016; 75: 811–818. 26269398 10.1136/annrheumdis-2015-207507

[48] Chan A Rigler K Rossen L , et al. Improving care and capacity through capturing and recording patient reported outcomes with digital solutions in spondyloarthritis. ACR 2021, Abstract 1788, https://acrabstracts.org/abstract/improving-care-and-capacity-through-capturing-and-recording-patient-reported-outcomes-with-digital-solutions-in-spondyloarthritis/

[49] Alivernini S Bruno D Tolusso B , et al. Differential synovial tissue biomarkers among psoriatic arthritis and rheumatoid factor/anti-citrulline antibody-negative rheumatoid arthritis. Arthritis Res Ther 2019; 21: 116. 31072400 10.1186/s13075-019-1898-7 PMC6509792

[50] Ørnbjerg LM Georgiadis S Jacobsson L , et al. Predictors of DAPSA28 remission at 6 months in bio-naive patients with psoriatic arthritis starting a TNF inhibitor in clinical practice-results from the eurospa collaboration. Arthritis Rheumatol 2019; 71: 2491.

[51] Chandran V Cook RJ Edwin J , et al. Soluble biomarkers differentiate patients with psoriatic arthritis from those with psoriasis without arthritis. Rheumatology (Oxford) 2010; 49: 1399–1405. 20421218 10.1093/rheumatology/keq105

[52] Magee C Jethwa H FitzGerald OM , et al. Biomarkers predictive of treatment response in psoriasis and psoriatic arthritis: a systematic review. Ther Adv Musculoskelet Dis 2021; 13: 1–14. 10.1177/1759720X211014010 PMC8111521 33995606

[53] Dixon WG Michaud K . Using technology to support clinical care and research in rheumatoid arthritis. Curr Opin Rheumatol 2018; 30: 276–281. 29369089 10.1097/BOR.0000000000000485 PMC5895111

[54] Bell S Merola JF Webster DE , et al. Aiming for cure and preventive initiatives in psoriatic disease: building synergy at NPF, GRAPPA, and PPACMAN. Curr Rheumatol Rep 2020; 2122: 78. 10.1007/s11926-020-00958-9 32959152

[55] Giovannini I Bosch P Dejaco C , et al. The digital way to intercept psoriatic arthritis. Front Med (Lausanne) 2021; 8: 792972. 34888334 10.3389/fmed.2021.792972 PMC8650082

[56] Najm A Nikiphorou E Kostine M , et al. EULAR points to consider for the development, evaluation and implementation of mobile health applications aiding self-management in people living with rheumatic and musculoskeletal diseases. RMD Open 2019; 5: e001014. 10.1136/rmdopen-2019-001014 PMC6744072 31565245

[57] Fagni F Knitza J Krusche M , et al. Digital approaches for a reliable early diagnosis of psoriatic arthritis. Front Med (Lausanne) 2021; 8: 718922. 34458293 10.3389/fmed.2021.718922 PMC8385754

[58] Scher JU Ogdie A Merola JF , et al. Preventing psoriatic arthritis: focusing on patients with psoriasis at increased risk of transition. Nat Rev Rheumatol 2019; 15: 153–166. 30742092 10.1038/s41584-019-0175-0

[59] Rahman P Elder JT . Genetics of psoriasis and psoriatic arthritis: a report from the GRAPPA 2010ual meeting. J Rheumatol 2012; 39: 431–433. 22298274 10.3899/jrheum.111242 PMC3779871

[60] Perez-Chada LM Haberman RH Chandran V , et al. Consensus terminology for preclinical phases of psoriatic arthritis for use in research studies: results from a Delphi consensus study. Nat Rev Rheumatol 2021; 17: 238–243. 33589818 10.1038/s41584-021-00578-2 PMC7997804

[61] Zabotti A De Lucia O Sakellariou G , et al. Predictors, risk factors, and incidence rates of psoriatic arthritis development in psoriasis patients: a systematic literature review and meta-analysis. Rheumatol Ther 2021; 8: 1519–1534. 34596875 10.1007/s40744-021-00378-w PMC8572278

[62] Gisondi P Bellinato F Targher G , et al. Biological disease-modifying antirheumatic drugs may mitigate the risk of psoriatic arthritis in patients with chronic plaque psoriasis. Ann Rheum Dis 2022; 81: 68–73. 34144965 10.1136/annrheumdis-2021-219961

[63] Meer E Merola JF Fitzsimmons R , et al. Does biologic therapy impact the development of PsA among patients with psoriasis? Ann Rheum Dis 2022; 81: 80–86. 34615637 10.1136/annrheumdis-2021-220761

[64] Merola JF Ogdie A . Does psoriasis treatment affect PsA development? Nat Rev Rheumatol 2021; 17: 708–709. 34635848 10.1038/s41584-021-00706-y

[65] Zabotti A Tinazzi I Aydin SZ , et al. From psoriasis to psoriatic arthritis: insights from imaging on the transition to psoriatic arthritis and implications for arthritis prevention. Curr Rheumatol Rep 2020; 22: 24. 32418006 10.1007/s11926-020-00891-x PMC7230038

[66] Cretu D Gao L Liang K , et al. Differentiating psoriatic arthritis from psoriasis without psoriatic arthritis using novel serum biomarkers. Arthritis Care Res (Hoboken) 2018; 70: 454–461. 28586166 10.1002/acr.23298

